# Reducing neuroinflammation by delivery of IL‐10 encoding lentivirus from multiple‐channel bridges

**DOI:** 10.1002/btm2.10018

**Published:** 2016-07-19

**Authors:** Daniel J. Margul, Jonghyuck Park, Ryan M. Boehler, Dominique R. Smith, Mitchell A. Johnson, Dylan A. McCreedy, Ting He, Aishani Ataliwala, Todor V. Kukushliev, Jesse Liang, Alireza Sohrabi, Ashley G. Goodman, Christopher M. Walthers, Lonnie D. Shea, Stephanie K. Seidlits

**Affiliations:** ^1^ Dept. of Biomedical Engineering Northwestern University Evanston IL, 48109; ^2^ Dept. of Biomedical Engineering University of Michigan Ann Arbor MI, 48109; ^3^ Dept. of Chemical and Biological Engineering Northwestern University Evanston IL, 48109; ^4^ Dept. of Bioengineering University of California Los Angeles Los Angeles CA, 90095; ^5^ Dept. of Chemical Engineering University of Michigan Ann Arbor MI, 48109; ^6^ Brain Research Institute University of California Los Angeles Los Angeles CA, 90095; ^7^ Jonsson Comprehensive Cancer Center University of California Los Angeles Los Angeles CA, 90024

**Keywords:** immune modulation, gene delivery, multiple‐channel bridge, spinal cord injury

## Abstract

The spinal cord is unable to regenerate after injury largely due to growth‐inhibition by an inflammatory response to the injury that fails to resolve, resulting in secondary damage and cell death. An approach that prevents inhibition by attenuating the inflammatory response and promoting its resolution through the transition of macrophages to anti‐inflammatory phenotypes is essential for the creation of a growth permissive microenvironment. Viral gene delivery to induce the expression of anti‐inflammatory factors provides the potential to provide localized delivery to alter the host inflammatory response. Initially, we investigated the effect of the biomaterial and viral components of the delivery system to influence the extent of cell infiltration and the phenotype of these cells. Bridge implantation reduces antigen‐presenting cell infiltration at day 7, and lentivirus addition to the bridge induces a transient increase in neutrophils in the spinal cord at day 7 and macrophages at day 14. Delivery of a lentivirus encoding IL‐10, an anti‐inflammatory factor that inhibits immune cell activation and polarizes the macrophage population towards anti‐inflammatory phenotypes, reduced neutrophil infiltration at both day 7 and day 28. Though IL‐10 lentivirus did not affect macrophages number, it skewed the macrophage population toward an anti‐inflammatory M2 phenotype and altered macrophage morphology. Additionally, IL‐10 delivery resulted in improved motor function, suggesting reduced secondary damage and increased sparing. Taken together, these results indicate that localized expression of anti‐inflammatory factors, such as IL‐10, can modulate the inflammatory response following spinal cord injury, and may be a key component of a combinatorial approach that targets the multiple barriers to regeneration and functional recovery.

## 1 Introduction

To date, the only treatment for treating spinal cord injury (SCI) with any degree of success in clinical trials is methylprednisolone, which mitigates the inflammatory response after injury.^1^ Inflammation after SCI initiates a damaging secondary injury response that establishes multiple barriers (e.g., accumulation of myelin debris, and reactive gliosis) to regeneration and restoration of function. The inflammatory cascade begins with upregulated production of inflammatory cytokines, chemokines, and reactive oxygen species (ROS),^2^, ^3^, ^4^, ^5^ which leads to recruitment of peripheral immune cells^6^ and activation of local microglia, oligodendrocytes, and astrocytes.^7^, ^8^, ^9^, ^10^ Early recruitment of neutrophils (PMNs)—and later macrophages—comprises the innate immune response to SCI. PMNs and macrophages recruited to the injury can remove pathogens; however, they also propagate secondary injury, which results in death of neurons and oligodendrocytes.^11^, ^12^ Macrophages, in particular, also phagocytose debris left behind by necrotic or apoptotic cells, including inhibitory myelin debris whose slow clearance contributes to the inability of axons to regenerate.^13^, ^14^ In addition, inflammatory cytokines produced by PMNs and macrophages induce astrocytes to adopt a reactive state in which they proliferate and can increase the secretion of chondroitin sulfate proteoglycans (CSPGs). These reactive astrocytes then form an inhibitory glial scar that surrounds the injury site and prevents regenerating axons from crossing.^9^, ^14^, ^15^, ^16^, ^17^, ^18^, ^19^

In normal wound healing, the inflammatory response resolves over time, leaving behind regenerated, functional tissue. In contrast, SCI results in a chronic immune response characterized by the persistence of inflammatory cells, insufficient clearance of cellular debris at the injury, and formation of a robust glial scar that creates a barrier that limits regeneration.^12^, ^20^, ^21^ In peripheral tissues, a transition in macrophage phenotype from inflammatory (M1 or classically activated) to anti‐inflammatory (M2 or alternatively activated) is largely responsible for dampening and resolving the immune response after injury.^22^, ^23^, ^24^ However, this transition does not occur on a sufficient scale after SCI. Instead, M1 macrophages chronically persist in zones of axon degeneration, where they propagate inflammation and inhibit axon regeneration.^25^, ^26^, ^27^, ^28^, ^29^, ^30^ Though a preponderance of evidence has shown that M1 macrophages predominate within the injury site after SCI, M2 macrophages are present—typically peaking at day 14 after injury in rodents.^31^ M2 macrophages generally have enhanced phagocytic capabilities^32^, ^33^ and can promote axon growth across inhibitory boundaries *in vitro*.^25^ They stimulate tissue repair through attenuated production of inflammatory cytokines, reduced ROS production, and expression of pro‐resolving cytokines such as IL‐10.^34^ In contrast, M1 macrophages are characterized by increased production of pro‐inflammatory cytokines, ROS and NO—each of which leads to neuron and oligodendrocyte toxicity.^25^, ^35^ Several factors promote M2 phenotypes including IL‐4, IL‐13, glucocorticoids, and IL‐10. Of these, IL‐10 was selected for this study due to its ability to promote neuron survival while reducing leukocyte infiltration and activation.^36^, ^37^, ^38^

Macrophage depletion has been investigated as a potential solution to mitigate the inflammatory response, but there have been mixed results. Treatment with liposome‐encapsulated clodronate to deplete hematogenous macrophages improved partial hindlimb recovery and tissue repair in one study^39^ and decreased fibrotic scarring while increasing axon numbers in a second study.^40^ In contrast, antibody‐mediated depletion of CD11c^+^ monocytes/macrophages or conditional ablation by diphtheria toxin each reduced functional recovery.^41^ These seemingly contradictory results suggest that specific macrophages phenotypes may have different effects on tissue repair and that eliminating or enhancing specific subtypes may be more beneficial than targeting the entire macrophage population. Additionally, there may be unintended systemic or local consequences of depleting the entire population, such as increased susceptibility to infection and disease and reduced clearance of inhibitory debris.

While macrophage phenotype has often been depicted as a binary system where a cell is either M1 or M2 at any given time, it is now thought that macrophages exist along a continuous spectrum of activation states, with M1 and M2 as polar opposites.^23^, ^42^ For example, some reports have sub‐divided M2 macrophages into additional subtypes (e.g., M2a, M2b, and M2c), each of which has a role in suppressing inflammation. The M2a (alternative) macrophages are involved in initial wound healing and the Th2‐type (T‐cell mediated) inflammatory response, while M2b (type 2) macrophages are believed to be immunoregulatory. Finally, M2c (deactivated) macrophages are immunosuppressive and facilitate matrix deposition and issue remodeling.^43^, ^44^ Despite observations of these macrophage subtypes *in vitro*, it remains unclear how this correlates with macrophage activation *in vivo*. Macrophages retain inherent capacity for plasticity along this activation spectrum, dynamically responding to changes in their local environment.^36^, ^45^ This dynamic plasticity makes macrophages a compelling target for resolving inflammation in the spinal cord to enable regeneration.

In addition to addressing neuroinflammation, therapies for spinal cord regeneration must provide physical support and guidance for regenerating axons across the injury site. SCI therapies focused solely on resolution of neuroinflammation have largely failed due to both lack of a growth‐promoting substrate and accumulation of inhibitory factors at the injury.^46^, ^47^ While immunomodulatory strategies can reduce inflammation and promote axonal sparing, guidance of regenerating axons requires a permissive substrate. Initial attempts to provide a permissive bridge across spinal cord lesions implanted donor peripheral nerve grafts.^48^ More recently, synthetic multiple‐channel bridges have been developed to replicate the synergistic advantages of peripheral nerve grafts—physical support and guidance and a biologically active, pro‐regenerative microenvironment.^49^, ^50^, ^51^, ^52^, ^53^, ^54^ We have previously demonstrated that multiple‐channel bridges made from biodegradable poly(lactide‐co‐glycolide (PLG) support robust crossing of axons across the lesion site, resulting in formation of regenerated axon bundles after complete bridge degradation 6 months after SCI.^54^, ^55^ Longitudinal, macroscale channels within these bridges act as conduits for axons regenerating across the injury site, while scaffold microporosity supports host cell infiltration and tissue integration.^55^, ^56^

Herein, we investigate the hypothesis that localized lentiviral expression of IL‐10 from multiple‐channel PLG bridges will modulate the numbers, phenotypes, and proportions of leukocyte populations infiltrating the bridge to promote a “resolving” anti‐inflammatory environment thought to be more permissive to regeneration. In addition to providing a substrate for regeneration, PLG bridges have also served as a platform for localized delivery of gene therapy vectors that can induce the expression of therapeutic proteins.^54^, ^57^ Biomaterial‐mediated lentivirus delivery after SCI efficiently transduces infiltrating cells to yield a localized, stable pattern of gene expression.^54^, ^58^ Lentiviral particles associated with the heparin‐modified PLG bridges, which functions to retain the vector locally and can increase its half‐life for enhanced gene transfer. Infiltrating host cells are transduced by the lentivirus, with peak transgene expression occurring approximately 7 days after bridge transplantation with sustained expression for at least 8 weeks.^54^, ^58^ Expression of IL‐10 will be investigated due to its neuroprotective and anti‐apoptotic properties, as well as its ability to skew macrophages towards anti‐inflammatory phenotypes.^36^, ^59^ The studies focus on the first four weeks after injury comprising the acute, subacute, and intermediate phases of recovery, which encompasses the time over which many of the barriers to regeneration become established.

## 2 Materials and methods

### 2.1 Virus production

Lentivirus was produced by co‐transfecting HEK‐293T cells with third generation lentiviral packaging vectors (pMDL‐GagPol, pRSV‐Rev, pIVS‐VSV‐G,^60^ and the gene of interest (pLenti‐CMV‐Luciferase or pLenti‐CMV‐IL‐10) using Lipofectamine 2000 (Life Technologies, Grand Island, NY, USA). After, lentiviral particles were purified using the Lenti‐X Maxi Purification Kit (Clontech Laboratories, Mountain View, CA, USA) and then concentrated using Vivaspin centrifugal concentrators (Sartorius, Göttingen, Germany). Viral titers used throughout the study were 2E10 IU/ml as determined by the Lentivirus qPCR Titer Kit (Applied Biological Materials, Richmond, BC, Canada).

### 2.2 Fabrication of multiple‐channel bridges

Bridges were fabricated using a sacrificial template variation^61^ of the gas foaming/particulate leaching technique,^54^ as previously described.^62^ Briefly, PLG (75:25 lactide:glycolide; inherent viscosity 0.76 dl/g; Lakeshore Biomaterials, Birmingham, AL, USA) was dissolved in dichloromethane (6% w/w) and emulsified in 1% poly(vinyl alcohol) using a homogenizer (PolyTron 3100; Kinematica AG, Littau, Switzerland) at 3000 rpm to create microspheres (z‐average diameter ∼1µm). D‐sucrose was caramelized, cooled, and drawn from solution with a Pasteur pipette to make sugar fibers. These fibers were coated with a mixture of PLG microspheres and salt (63–106 μm) and pressed into a salt‐lined aluminum mold. The materials were then equilibrated with CO_2_ gas (800 psi) for 16 hrs and then gas foamed in a custom‐made pressure vessel. Bridges were subsequently cut into 2.25 mm sections and leached for 2 hrs to remove porogens. The bridges are dried overnight and stored in a desiccator.

### 2.3 Heparinization of nerve bridges

Heparin coating of bridges has been shown to enhance lentiviral loading and transduction from bridges *in vivo*.^63^ PLG bridges were incubated with chitosan (Sigma Aldrich, St. Louis, MO, USA; 25 μg/μl in 2% glacial acetic acid) for 10 min, followed in a N‐(3‐dimethylaminopropyl)‐N′‐ethylcarbodiimide/N‐hydroxylsuccinimide (Sigma Aldrich) mixture dissolved in 2‐(N‐morpholino)ethanesulfonic acid (EDC/NHS in MES; 1.5:1:1 mg/mg/ml, Sigma Aldrich) for 2 hrs. To conjugate chitosan, bridges were washed 3 times with water, dried and subsequently incubated with heparin (Sigma Aldrich; 25 μg/μl in 1M MES) for 10 min followed by EDC/NHS in MES for an additional 2 hrs for covalent conjugation. Finally, bridges were washed in water three times and dried.

### 2.4 Virus loading onto heparinized nerve bridges

Multiple additions of viruses were adsorbed onto bridges in an iterative manner in order to increase lentiviral loading. Prior to virus addition, bridges were disinfected in 70% ethanol and washed with water. After 12 min of drying time, bridges were saturated with 2 μl of virus. Bridges were then dried for an additional 12 min followed by another 2 μl of virus. Bridges were then dried for 14 additional min followed by a final 2 μl of virus. After a final 5 min of drying, bridges were stored at −80°C until used for surgery.

### 2.5 Mouse spinal cord hemisection

A hemisection model of SCI was performed as previously described^62^ on female C57/BL6 mice (4‐6 weeks‐old; Charles River Laboratories, Wilmington, MA, USA), according to the Animal Care and Use Committee guidelines at Northwestern University. A laminectomy was performed at T9‐T10 to allow for a 2.25 mm lateral hemisection for bridge implantation. The injury site was covered using Gelfoam (Pfizer, New York, NY, USA) followed by suturing together of the muscle and stapling of skin. Postoperative care consisted of administration of enrofloxacin (2.5 mg/kg; daily for 2 weeks), buprenorphine (0.1 mg/kg; twice daily for 3 days), and Lactated Ringer's solution (5 ml/100 g; daily for 5 days). Bladders were expressed twice daily until function recovered.

### 2.6 Tissue processing and immunofluorescence

Spinal cord tissue was collected at days 7, 14, and 28, which were chosen to represent acute, subacute and intermediate phases of regeneration respectively. It may be noted that days 7 and 14 are at the tail end of their respective phases; however, it is not possible to extract the spinal cord and bridge together prior to day 7 as the bridge falls out due to a weak interface tissue‐bridge interface. Additionally, cellular infiltration is lower at earlier time points, making flow cytometry challenging. For immunofluorescence, spinal cord segments were then snap frozen in isopentane and embedded in Tissue Tek O.C.T. Compound (Sakura Finetek, Torrance, CA, USA) with 20% sucrose. Cords were cryo‐sectioned transversely in 18‐μm‐thick sections.

Antibodies against the following antigens were used for immunofluorescence: neurofilament 200 (NF200, Sigma Aldrich), myelin basic protein (MBP, Santa Cruz Biotech, Dallas, TX, USA) P‐zero myelin protein (P0) (Aves Labs, Tigard, OR) F4/80 (AbD Serotec, Raleigh, NC, USA) arginase I (clone N20, Santa Cruz Biotech) and Ly‐6G (clone RB6‐8C5, Biolegend).

Using stained tissue sections, immuno‐positive cells and axons within the bridge area were quantified manually by two blinded researchers independently. Co‐staining for multiple markers was assessed by evaluating pixel overlap of different channels in NIH ImageJ (Bethesda, MD, USA). Total numbers of F4/80^+^ cells and numbers of F4/80^+^/arginase^+^ cells were evaluated to determine numbers of total macrophages and M2 macrophages, respectively. Macrophages were identified by localizing Hoechst nuclear staining to F4/80^+^ immunofluorescence and labeling them in ImageJ. Subsequently, each identified F4/80^+^ cell was binned based on having arginase immunofluorescence visible over background. Cell counts were then normalized to counted area in each tissue section. Macrophage shape was characterized for each positive cell and cells were binned as fibrous/elongated, round and not ruffled with a bright F4/80^+^ perimeter, or as multinucleated foreign body giant cell (FBGC). To assess the numbers of regenerated and myelinated axons, NF200 was used to identify axons, NF200^+^/MBP^+^ to determine the number of myelinated axons, and NF200^+^/MBP^+^/P0^+^ to determine the amount of myelin derived from infiltrating Schwann cells.^57^

### 2.7 Flow cytometry of digested tissue samples

Flow cytometry was performed on spinal cord bridge implants loaded with no lentivirus, lentivirus encoding firefly luciferase (FLUC) or IL‐10 to determine the identity of infiltrating cells 7, 14, and 28 days after injuries. Spinal cord hemisection with no bridge implants were also performed. To isolate sufficient cell numbers for flow cytometry, implant sites from 3 individual mice were pooled together per condition. Bridge tissue samples were mechanically dissociated by 16g needle and syringe and digested in a solution containing collagenase (1 mg/ml; Worthington Biochemical Corp., Lakewood, NJ, USA) and trypsin (0.5 mg/ml; Life Technologies) for 20 min at 37°C. After incubation, 1 ml of fetal bovine serum (Sigma Aldrich) was immediately added to inhibit digestion and solution was triturated with fire‐polished glass pipettes of at least three successively decreasing diameters to achieve a single cell suspension. Digested tissues were pushed through 70 μm strainers and washed with Hank's Balanced Salt Solution without calcium and magnesium (VWR, Radnor, PA, USA). Cells were then separated from myelin using the OptiPrep gradient system (Sigma Aldrich) in MOPS buffer, as previously described by Beck et al.^20^ Cells were subsequently blocked with a solution containing 1% normal mouse and rat serum (Sigma Aldrich) and anti‐mouse CD16/32 (eBioscience) and stained for viability using fixable violet dead cell stain (Invitrogen) and stained with the specific antibodies listed above. Data were acquired on a BD LSR II cytometer, and analyzed using FloJo software. Fluorescence minus one staining was used as a negative control.

The following flow cytometry antibodies were used: v500‐conjugated 30‐F11 against CD45, PE(phycoerythrin)/Cy7‐conjugated RM4‐5 against CD4, and Alexa Fluor 647‐conjugated 1B4 against GFAP (glial fibrillary acidic protein) (BD Biosciences, San Jose, CA, USA), PE‐conjugated N418 against CD11c, APC/eFluor780‐conjugated BM8 against F4/80 (eBioscience, San Diego, CA, USA), PerCP‐conjugated RB6‐8C5 against Gr‐1 (high affinity Ly‐6G, low affinity Ly‐6C,^64^, ^65^ Biolegend, San Diego, CA, USA). Unconjugated antibody against CNPase (2′,3′‐cyclic‐nucleotide 3′‐phosphodiesterase) was purchased from Abcam and conjugated to fluorescein isocyanothionate (FITC) using an EasyLink Conjugation kit (Abcam, Cambridge, MA, USA).

### 2.8 mRNA isolation and qRT‐PCR analysis

To isolate mRNA, spinal cord tissues and bridge implants were explanted with 2 mm of spinal cord both rostral and caudal to the bridge. Explanted tissues were homogenized using 1 ml of Trizol reagent (Life Technologies) with a tissue grinder. RNA isolation was followed by chloroform extraction and isopropanol precipitation.^66^ The extracted RNA was dissolved in 30 μ*l* of RNase‐free distilled water and RNA concentration was measured using a NanoDrop 2000C (ThermoFisher Scientific, Newark, DE, USA) and to assure sufficient purity (A260/A280 ratios between 1.9 and 2.1 for all samples). Total isolated RNA was stored at −80°*C* freezer until use. cDNA was synthesized using iScript™ cDNA Synthesis kit (Bio‐Rad, Hercules, CA, USA) according to the manufacturer's instructions using 1 µg of RNA per sample.

Primers for qRT‐PCR (quantitative real‐time polymerase chain reaction) quantification of arginase I expression were chosen based on a previous study^25^: forward 5′‐GAACACGGCAGTGGCTTTAAC‐3′, and reverse 5′‐ TGCTTAGCTCTGTCTGCTTTGC‐3′. 18s‐rRNA was used as an internal control with following sequences: forward 5′‐GCAATTATTCCCCATGAACG‐3′, and reverse 5′‐ GGCCTCACTAAACCATCCAA‐3′.^67^ The qRT‐PCR products were measured using the accumulation level of iQ™ SYBR Green Supermix (Bio‐Rad) fluorescence following a manufacturer's protocol on CFX Connect™ Real‐Time PCR Detection System (Bio‐Rad). The gene expression level of arginase I mRNA was normalized to that of 18s‐rRNA and differences in gene expression were presented as fold ratios from sham (laminectomy only) spinal cord samples. Relative quantification was calculated as *X* = 2^−ΔΔ^ ^*C*^ ^t^, where ΔΔ*C*_t_ = Δ*E*−Δ*C*_t_ and Δ*E* = *C*_t,exp_ – *C*_t,18s‐rRNA_, Δ*C*_t_ = *C*_t, sham_ – *C*_t,18s‐rRNA._ 68

### 2.9 Behavioral analysis

The Basso mouse scale (BMS) open‐field locomotor test (range 0–9) was used to assess functional recovery for a period 24 weeks after SCI as previously described.^69^ A baseline was determined prior to SCI, and mice were tested 3, 7, 14, 21, and 28 days. Observations and BMS scoring were performed by two trained observers at 4‐min intervals.

### 2.10 Statistical analysis

For multiple comparisons, statistical significance between groups was determined by one‐way or two‐way ANOVA with Tukey's post hoc testing or Šidák post hoc testing. For single comparisons, the statistical significance between pairs was determined by unpaired *t* test. For, statistical analysis of macrophage histology, data sets were standardized using an ln(*x*+1) transformation to eliminate skewness and normalize to a Gaussian distribution as verified with D'Agostino‐Pearson omnibus test.^70^ All statistics test significance using a *p* value of .05 unless otherwise noted. Error bars represent standard error in all figures. Prism 7 (GraphPad Software, La Jolla, CA, USA) software was used for all data analysis.

## 3 Results

### 3.1 Cell infiltration into bridges

**Figure 1:**
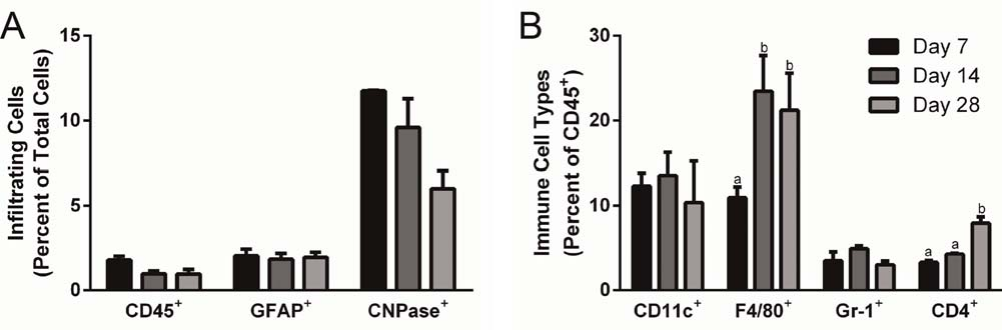
Time course of immune cell infiltration into spinal cord bridges. Infiltrating cells form the central nervous system and peripheral immune system were identified in bridges implanted into a hemisection spinal cord injury (*n* = 6–21 mice) (**A**) CD45^+^ immune cell, GFAP^+^ astrocyte, and CNPase^+^ oligodendrocyte infiltration into bridges were quantified as a percentage of total cells using flow cytometry at days 7, 14, and 28. (**B**) Immune sub‐populations, including CD11c^+^ dendritic cells, F4/80^+^ macrophages, Gr‐1^+^ neutrophils, and CD4^+^ helper T cells, in the bridge were quantified as a percentage of total immune cells using flow cytometry at days 7, 14, and 28. Statistical analysis completed using a one‐way ANOVA with Tukey's post hoc test. Significantly different groups denoted by letter with “a” and “b” denoting statistical significance between the two groups (*p* <.05)

Cell infiltration into the bridges was initially investigated to identify the cell populations and their abundance at multiple time points. No statistically significant changes in the numbers of CD45^+^ immune cells or GFAP^+^ reactive astrocytes were observed within the bridges between days 7 to 28 (Figure 1A). Additionally, although no significant decrease in CNPase^+^ oligodendrocytes was observed during this time, a trend towards decreasing abundance was seen (Figure 1A). When sub‐populations of CD45^+^ immune cells were assessed, a significant increase in the percentage of F4/80^+^ macrophages was observed from day 7 to day 14 (Figure 1B**)**. Numbers of Gr‐1^+^ neutrophils (PMNs) and CD11c^+^ dendritic cells (DCs) did not significantly change over time. Finally, CD4^+^ helper T (T_H_) cells had a significant increase in abundance from days 14 to 28, indicating that the adaptive immune response is activated at later time points.

### 3.2 IL‐10 gene delivery from bridges reduces PMN infiltration

**Figure 2:**
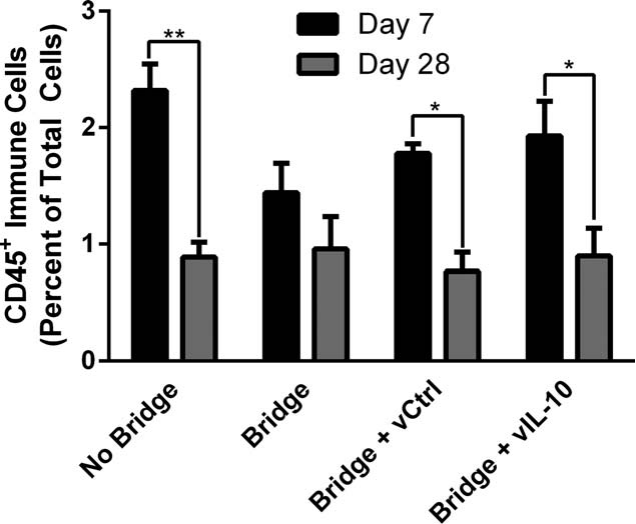
Bridge implantation and lentivirus delivery do not increase immune cell infiltration. The infiltration of immune cells into the injury site after hemisection spinal cord injury were quantified as a percentage of total cells by flow cytometry at days 7 and 28 (*n* = 9–15 mice). A mock implantation consisting of the hemisection injury without bridge implantation (no bridge) was compared to bridge implantation, luciferase control lentivirus (vCtrl) loaded bridge implantation, and IL‐10 lentivirus (vIL‐10) loaded bridge implantation. Statistical analysis was completed using a two‐way ANOVA with a Šidák correction for multiple comparisons. Significant differences between days 7 and 28 notated with ** (*p* <.01) or * (*p* <.05)

**Figure 3:**
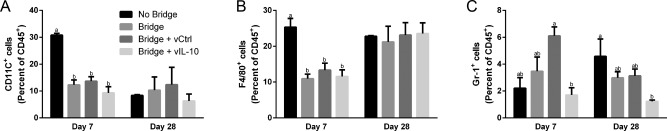
IL‐10 delivery from bridge reduces neutrophil infiltration. The infiltration of immune cell sub‐populations into the injury site was investigated for no bridge, bridge, luciferase control lentivirus (vCtrl) loaded bridges, and IL‐10 lentiviral (vIL‐10) loaded bridges (*n* = 6–21 mice). Infiltration of sub‐populations of innate immune cells were quantified by flow cytometry at day 7 and day 28 including (A) CD11c^+^ dendritic cells, (B) F4/80^+^ macrophages, and (C) Gr‐1^+^ neutrophils. Statistical analysis completed using a one‐way ANOVA with Tukey's post hoc test. Significantly different groups denoted by letter with “a” and “b” denoting statistical significance between the two groups *p* < .05 for Gr‐1 and *p* < .005 for all others

The immune response to bridges with and without delivery of IL‐10‐encoding lentivirus was investigated to determine if the infiltration of inflammatory cells can be modulated through localized cytokine expression. In previous reports, we have demonstrated lentiviral IL‐10 expression^36^ and sustained transgene expression for at least 8 weeks in the injured spinal cord.^63^ The flow cytometry studies were performed at days 7 and 28, to capture differences from the acute to intermediate phases of recovery observed in Figure 1. First, though differences in infiltrating immune cells (CD45^+^) as a percentage of total cells were not statistically different at day 7, the addition of a bridge to the SCI resulted in a 38% decrease and the addition of lentivirus to the bridge resulted in a 23–34% increase over the bridge alone (Figure 2). This result indicates that the bridge may participate in stabilizing the injury and reducing inflammation at early time points. From day 7 to 28, hemisection alone (no bridge implantation) and bridges loaded with either lentivirus had a reduction of greater than 50% (*p* < .05) in CD45^+^ cells as a percentage of total cells. Bridges without lentivirus had only a 33% reduction over this same time. At day 28, immune cells as a percentage of total cells were similar for all conditions. Subpopulations of CD45^+^ immune cells were characterized at days 7 and 28 with all bridge conditions having a 2‐fold decrease in the percentage of CD11c^+^ antigen‐presenting cells (APCs, including dendritic cells and macrophages) relative to no bridge implantation at day 7 **(**Figure 3A**)**. This reduction did not persist to day 28, at which point APC numbers were similar across all conditions. Interestingly, the hemisection‐only condition had a dramatic 3.5‐fold (*p* < .01) decrease in APC infiltration. The percentage of infiltrating F4/80^+^ macrophages as percent of CD45^+^ cells was reduced approximately 2‐fold in the bridge conditions relative to the no bridge control at day 7; however, by day 28, the macrophage percentage increased in the bridge conditions such that they were no longer significantly less than the no bridge condition **(**Figure 3B**)**. Notably, these changes represent the percentage of CD45^+^ cells that were also F4/80^+^; thus, the increase in macrophage percentage at day 28 does not imply an increase in the total number of macrophages. Finally, delivery of IL‐10‐encoding lentivirus reduced numbers of infiltrating PMNs by 3.5‐fold when compared to the bridges loaded with control lentivirus at Day 7 (*p* < .05) (Figure 3C). Bridges loaded with IL‐10‐encoding lentivirus had similar numbers of infiltrating PMNs as in hemisections with no bridge implant. Localized IL‐10 expression at the bridge did sustain a 3.5‐fold reduction of PMN infiltration through day 28 (relative to the no bridge control) (*p* < .05) (Figure 3C). Additionally, IL‐10 expression reduced the mean level of PMN infiltration by 2.4‐fold compared to all other bridges, though this decrease was not statistically significant (Figure 3C).

### 3.3 IL‐10 gene delivery from bridges increases M2 macrophage infiltration

Immunofluorescence staining of tissue sections was applied to investigate morphological features and distribution of immune cells, which complements the flow cytometry characterization of the relative populations of immune cells infiltrating the bridge.

**Figure 4:**
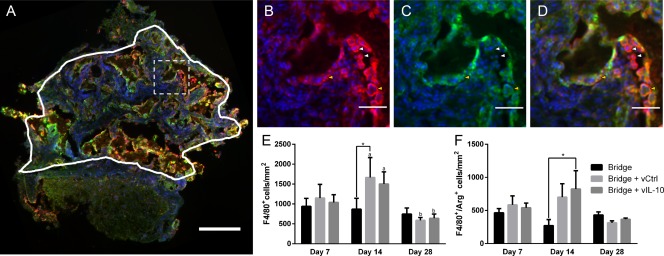
IL‐10 delivery increases the density of M2 macrophages. (A) Immunofluorescence (F4/80^+^/arginase^+^/Hoechst^+^: Red/Green/Blue, respectively) was used to characterize the phenotype of infiltrating macrophages within the bridge (scale bar 200 µm). Line indicates the bridge area used for quantification and dashed line indicates higher magnification regions in (B) F/480^+^ total macrophages (C) arginase^+^ cells, and (D) merge (scale bar 50 µm). Brightness and contrast were adjusted for clarity. Arrows note arginase^‐^ macrophages (white) and arginase^+^ macrophages (yellow). (E) Quantification of density (cells/mm^2^) of total F4/80^+^ macrophages as counted manually. (F) Quantification of density (cells/mm^2^) of F4/80^+^/arginase^+^ M2 macrophages as counted manually. For all quantification, mean +/− SEM was plotted and *n* ≥3 where each replicate is an average of 1–4 tissues from an individual animal. Statistical analysis was completed on log normalized data using a two‐way ANOVA with Tukey's post hoc test with statistical significance between groups indicated by a single asterisk (*p* <.05). Statistical significance of individual conditions between time points indicated by letter with “a” and “b” denoting statistical significance between the two groups (*p* < .05)

Immunofluorescently stained sections were initially analyzed to quantify densities of total macrophages (F4/80^+^) and M2 macrophages (F4/80^+^/arginase^+^) (Figure 4 **)**. A statistical difference (*p* < .05) was observed in macrophage density between days 14 and 28 for delivery of lentivirus (encoding FLUC or IL‐10) from the bridge. In agreement with the flow cytometry results, macrophage number was similar between days 7 and 28 for all conditions **(**Figure 4E**)**. We subsequently characterized the density of F4/80^+^ cells that also expressed arginase, a marker for the M2 macrophage phenotype. A significant increase in arginase^+^ macrophage density was observed at day 14 in the bridges loaded with IL‐10‐encoding lentivirus relative to control bridge implants (no lentivirus) **(**Figure 4F**)**, with a similar trend observed relative to FLUC loaded bridges.

**Figure 5:**
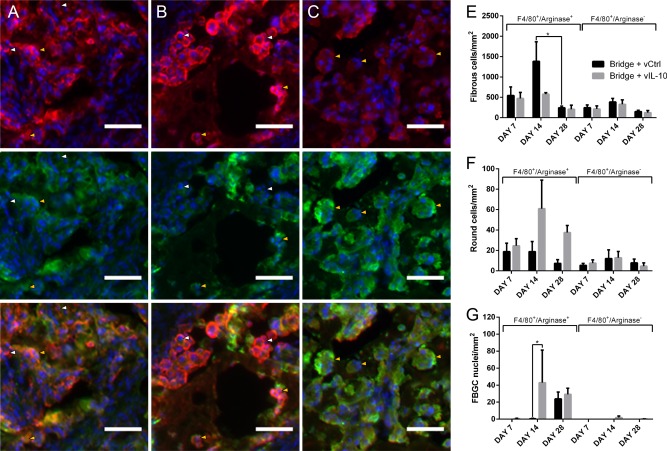
IL‐10 alters macrophage morphology. Immunofluorescence (F4/80^+^/arginase^+^/Hoechst^+^: red/green/blue, respectively), was used to characterize the morphology of infiltrating macrophages within the bridge as either (A) fibrous, (B) round, or (C) FBGC (scale bar 50 µm). Arrows note arginase^‐^ macrophages (white) and arginase^+^ macrophages (yellow). (D) Manual quantification of density (cells/mm^2^) of fibrous macrophages. (E) Manual quantification of density (cells/mm^2^) of round macrophages. (F) Manual quantification of density (fused nuclei/mm^2^) of FBGC macrophages. For all quantification, mean +/−SEM was plotted and *n* ≥3 where each replicate is an average of 1–4 tissues from an individual animal. Statistical analysis was completed on log‐normalized data using a two‐way ANOVA with Šidák correction for multiple comparisons with statistical significance indicated by a single asterisk (*p* < .05)

The morphology of the F4/80^+^ cells was subsequently characterized to further represent macrophage phenotype. The F4/80^+^ cells were categorized according three distinct morphologies. The first morphology consisted of fibrous, elongated cells that appeared to have multiple processes **(**Figure 5A**)**. A second morphology was round and had a prominent F4/80^+^ border, and a majority of these round cells were arginase^+^ **(**Figure 5B**)**. The third F4/80^+^ morphology consisted of multinucleated foreign body giant cells (FBGCs) **(**Figure 5C**),** and nearly every nucleus in the FBGCs was surrounded by arginase^+^ immunostaining. Each macrophage was binned according to arginase expression and morphology (fibrous, round, or FBGC). For the fibrous macrophages, the density was largely consistent across conditions and time points, with the singular exception of a 5.5‐fold decrease in density between days 14 and day 28 for the control virus condition **(**Figure 5D**)**. For the round macrophages, no significant differences were observed, though a strong trend (p = .088) toward an increase in arginase^+^ round macrophages was identified for IL‐10 expression relative to FLUC across all time points **(**Figure 5E**)**. Finally, for the FBGCs, a greater density of the arginase^+^ cells was observed at Day 14 for the IL‐10 conditions relative to the FLUC virus control **(**Figure 5F**)**.

### 3.4 IL‐10 delivery results in elevated arginase expression

**Figure 6:**
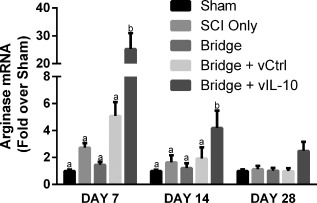
Il‐10 results in elevated arginase mRNA. qRT‐PCR revealed elevated arginase levels at 7 and 14 days after SCI. Levels statistically returned to baseline by day 28, but a strong trend remained (0.08 < *p* < 0.13). *N* = 4–6 per group for PCR. Statistical analysis was completed using a one‐way ANOVA with Tukey's post hoc test at each time point. Significantly different groups were denoted with letters with “a” and “b” denoting statistical significance between the two groups: day 7 (*p* <.001) and day 14 (*p* <.0001)

mRNA levels for arginase were measured by qRT‐PCR to complement the arginase^+^ cells density assessment by immunofluorescence. At day 7, qRT‐PCR revealed a 5‐fold or greater increase in arginase mRNA in the IL‐10 group (*p* < .001) **(**Figure 6 **)**. Increased mRNA at day 7 may correlate to increased protein observed in tissue sections at Day 14 (Figure 4F). At day 14, arginase expression in IL‐10 bridges was elevated almost 5‐fold compared to bridges loaded with FLUC‐encoding lentivirus (*p* < .0001). At day 28, arginase mRNA levels remained more than 2‐fold higher for the IL‐10 condition, but this difference was not statistically significant (.08 < *p* < .13)

### 3.5 Axon regeneration and myelination

**Figure 7:**
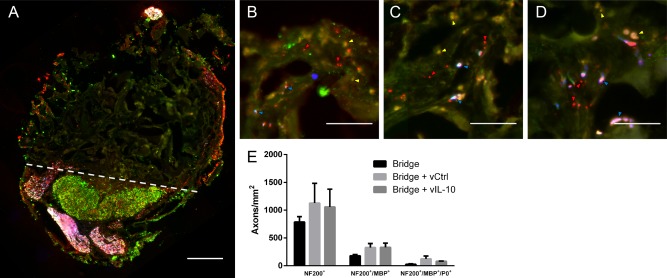
Myelinated axons 4 weeks after injury. (A) Immunofluorescence of myelinated (NF200^+^/MBP^+^/P0 red/green/blue, respectively) and total (NF200^+^: red) axons from bridge implants (scale bar 200 µm). Dashed line indicates border between bridge implant and host tissue. NF200^+^/MBP^+^ Immunofluorescence at higher magnification from bridges delivering (B) No virus (empty), (C) F‐LUC, or (D) IL‐10 (scale bar 50 µm). Brightness and contrast were adjusted for clarity. Arrows note unmyelinated axons (red), oligodendrocyte myelinated axons (yellow), and Schwann cell myelinated axons (blue). (E) Quantification of total axon numbers, total myelinated axons, and Schwann cell myelinated axons. (mean +/−SEM)

Axon numbers and myelination were quantified at day 28 to assess whether IL‐10 overexpression would prove detrimental, which has been reported in the peripheral nervous system.^71^ Myelinated (NF200^+^/MBP^+^) and unmyelinated (NF200^+^/MBP^‐^) axons were seen throughout the bridges in animals receiving no lentivirus, FLUC‐encoding control lentivirus, or IL‐10‐encoding lentivirus **(**Figure 7A–D**)** 28 days after SCI. NF200^+^ axons were typically observed as bundles as previously reported for multichannel PLG bridges.^54^, ^57^, ^72^ Empty bridges had approximately 800 neurites/mm^2^, and both lentiviral conditions had approximately 1100 neurites/mm^2^. While the data suggest a trend toward greater densities of regenerating axons with lentivirus delivery, these differences were not statistically significant. Similarly, the percentage of axons that were myelinated (22–34%) or the percentage of myelination that was derived from Schwann cells (15–35%) did not vary between conditions **(**Figure 7E**)**.

### 3.6 Motor function is improved with IL‐10

**Figure 8:**
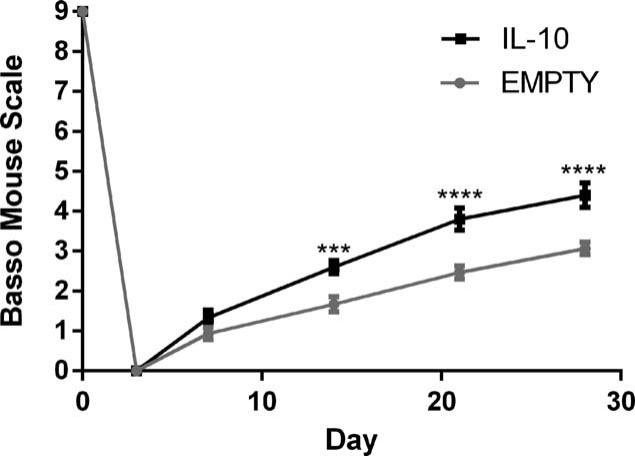
Functional recovery induced by IL‐10 overexpression. The BMS was used to test for differences in motor function in the hindlimb ipsilateral to SCI (*n* = 15 per group). Statistical analysis was completed using a two‐way ANOVA with repeated measures and a Šidák correction for multiple comparisons. Significant differences notated with *** (*p* < .001) or **** (*p* < .0001)

Motor function in the ipsilateral hindlimb was characterized over the first 28 days after SCI using the BMS (Figure 8 **)**. Prior to surgery, all mice had full function, and 3 days post injury, no movement was observed in the ipsilateral hindlimb of any mice. From day 14 onward, mice receiving IL‐10 lentivirus had substantially improved function in comparison mice that received a bridge without lentivirus. Mice receiving IL‐10 lentivirus received a BMS score of ∼4.4, where 4 indicates the capacity to take a step (defined as having weight support at lift off, forward limb advancement, and re‐establishment of weight support at initial contact) and 5 indicates that stepping occurs at least 50% of the time. In contrast, without IL‐10, mice scored an average of ∼3, which only indicates plantar placement of the foot while moving.

## 4 Discussion

The studies reported herein employ lentiviral delivery from bridges in order to induce sustained expression of the anti‐inflammatory cytokine IL‐10 to modulate the inflammatory response. Previous reports of lentiviral delivery from a bridge implanted into the injured spinal cord have demonstrated sustained expression for at least 8 weeks, with maximal expression localized within the bridge and decreased expression both rostral and caudal to the bridge.^60^, ^63^ IL‐10 has been reported in some studies to be neuroprotective, reduce secondary inflammation, and reduce lesion volume; however, others have suggested that IL‐10 has insignificant effects on spinal cord regeneration. This discrepancy in responses may result from the different models of SCI, or from differences in delivery. Notably, IL‐10 does not pass the blood‐brain barrier (BBB), and must be delivered either locally or pass through a disrupted BBB following injury. IL‐10 also is unstable with a short half‐life of 1.1–2.6 hrs, necessitating repeated doses or sustained expression approaches.^74^ Systemic overexpression of IL‐10 has been investigated, yet this approach is associated with peripheral neuropathy and demyelination of the sciatic nerve.^71^ Systemic delivery of IL‐10 protein has had conflicting results: either increasing tissue damage with impaired locomotion or reducing lesion size.^47^, ^75^ IL‐10 poliovirus elevated IL‐10 for 4 days with modestly improved function, and intrathecal injection of HSV‐encoding IL‐10 improved axonal sparing and functional recovery.^37^, ^38^ Despite several studies investigating IL‐10 delivery for SCI, the effects of sustained localized delivery on dampening and resolving the immune response to promote regeneration have not previously been studied.^59^ and are the focus of the studies herein.

We initially demonstrate that delivery of IL‐10‐encoding lentivirus can decrease PMN infiltration into biomaterial bridges implanted following SCI, and that though the loading of lentivirus to bridges may promote PMN infiltration, IL‐10 over‐expression reverses this effect. Although PMNs are transient participants in the immune response following injury to peripheral tissues, they can persist for weeks after injury to the spinal cord.^12^, ^20^ Furthermore, though virus delivery from biomaterials can be used to enhance nerve regeneration,^54^, ^76^ addition of virus to the biomaterial platform can increase the extent of cell infiltration.^77^, ^78^, ^79^ Secretion of chemokines from the increased immune cells may be responsible for the increase in PMN infiltration at day 7 and 28 reported here (Figure 3C), though increased cytokines would also be expected to simultaneously influence macrophage infiltration, which was not observed and is in agreement with a previous report.^58^ Due to their primarily bactericidal role in wound healing, PMNs are not expected to provide neuroprotection and are primarily detrimental in SCI.^12^ PMNs (and macrophages) release cytokines, free radicals, eicosanoids, and proteases, which are toxic to neurons and glia. In particular, superoxide, nitric oxide, and peroxynitrite are highly toxic and create irreversible damage to cellular components, inducing apoptosis.^80^ However, tail vein administration of the RB6‐8C5 Ly6G/Gr‐1 antibody, which selectively depleted the hematogenous PMN population by more than 90%, resulted in less glial scarring, decreased spared tissue, and worse BMS functional results.^81^ Cytokines released by neutrophils may be necessary for sufficient formation of reactive astrocytes to seal the injury and re‐establish the BBB. In contrast, reducing infiltration of PMNs (↓70%) and macrophages (↓36%) by administration of antibodies against CD11d/CD18 results in less ROS, decreased Caspase 3, and improved functional recovery.^82^, ^83^, ^84^ These results suggest that though PMNs are essential for the initiation of proper wound healing in the acute injury stage, strategies that mitigate PMN presence in later stages of injury may improve regeneration. Notably, the decrease reported herein regarding the chronic persistence of PMNs, a feature characteristic of mouse and human SCI, has not been previously reported^12^, ^20^, ^25^ These reports have demonstrated PMNs lasting through 180 days in mice and 12 months in humans.^20^, ^85^ Neutrophils are generally believed to aggravate SCI, though there may be some beneficial function that remains unidentified.^20^ Taken together, the sustained production of IL‐10 through lentivirus transduction alone, or preferably as part of a combinatorial therapy, may prove crucial to reducing the secondary damage and cell death associated with PMN infiltration^11^ and enhancing regeneration.

Macrophage phenotype, and to some extent number, was influenced by lentiviral delivery of IL‐10. Flow cytometry and immunofluorescence staining demonstrated no changes in F4/80^+^ macrophage numbers at day 7 and 28 between conditions. At day 14, quantification revealed a 1.7‐fold increase in the number of F4/80^+^ macrophages with expression of IL‐10 relative to the control conditions (*p* < .05). These results were expected, as macrophage number is known to peak around Day 7‐10 and plateau for several days before decreasing throughout the intermediate and chronic phase.^12^, ^20^ More importantly, IL‐10 production altered macrophage polarity with a substantial (3‐fold) increase in M2 (F4/80^+^/arginase^+^) macrophage numbers at Day 14 relative to empty bridges (*p* < .05). While the number of arginase^+^ macrophages was not elevated at Day 7 or Day 28, arginase mRNA was upregulated at Day 7 and 14, and trended upward at Day 28. Macrophages can have varying degrees of arginase expression as seen in Figure 4, which may depend on the specific subtype and phase of regeneration (inflammatory, proliferative, or remodeling).^44^ IL‐10 known for its ability to upregulate expression of IL‐4Rα, which may synergize with IL‐4 dependent arginase expression.^86^ Additionally, alternatively activated (M2) macrophages produce IL‐10, potentially creating a feed forward process when IL‐10 is overexpressed.

IL‐10 expression influenced the relative distribution of three macrophage morphologies: elongated, fibrous cells, round cells with a prominent F4/80 border, and FBGCs. The significance of these morphologies is unclear, and the literature has little information about the relationship between macrophage morphology and phenotype. *In vitro* studies, in particular, have been conflicting. Human macrophages polarized to M1/M2 with LPS/IFNγ and IL‐4 respectively that were cultured on tissue culture plastic or within collagen gels exhibited elongated morphologies for M1 macrophages and round, less adherent morphologies for M2 macrophages.^87^ In contrast, when C57BL/6 macrophages are cultured on fibronectin‐coated polydimethylsiloxane molds, M1 macrophages have a rounded morphology while M2 macrophages have a fibroblast‐like morphology.^88^ Moreover, McWhorter et al. demonstrated that substrates patterned with lines (width 20 µm) could elongate macrophages leading to upregulated arginase expression.

*In vivo* characterization of the relationship between macrophage phenotype and morphology has similarly been conflicting. One hypothesis is that the elongated F4/80^+^ cells are activated hematogenous macrophages, while the round cells are activated resident microglia. Microglia are well known to proceed from a highly ramified morphology toward an amoeboid morphology as they become activated and phagocytic after injury.^89^ Unfortunately, antibodies have not been identified to accurately distinguish macrophages from microglia using immunohistochemistry, though future studies using genetic models such as *cr2* ^rfp^::*Cx3cr1* ^gfp^ mice^90^ have the potential to distinguish cell morphology, phenotype, and lineage. Alternatively, these different morphologies may represent distinct M2 phenotypes. Shechter et al. selectively ablated monocyte‐derived macrophages followed by adoptive transfer of monocytes.^41^ The exogenous monocytes differentiated into macrophages with an activated morphology, which was manifested by a large cell body with few to no processes, arginase expression, and IL‐10 release. These factors suggested an anti‐inflammatory phenotype, and the monocytes contributed to regeneration.^41^ A subset of infiltrating monocyte‐derived, anti‐inflammatory macrophages may be essential for recovery, though these cells represent a specific subset of M2 macrophages and will require further characterization.^91^, ^92^

The round macrophages with prominent F4/80^+^ borders may be undergoing fusogenesis. FBGCs have not been extensively studied in the spinal cord and their impact on spinal cord regeneration is unknown. Fusion has been proposed to begin with the creation of fusion‐competent macrophages by IL‐4/IL‐13 and/or DAP12/TREM‐2 interactions between adjacent macrophages,^93^ followed by CCL2‐mediated chemotaxis and E‐cadherin‐based macrophage adhesion. Despite this simple model, the general requirements for fusion are not fully understood in that it is not clear what role phagocytic capability, macrophage subtype, and material interactions play in their formation.^93^ Additionally, though IL‐4 and IL‐13 are typically thought of as cytokines associated with macrophage fusion,^93^ IL‐10 co‐localizes with M2 macrophages at sites of macrophage fusion during FBGC development.^94^ Whether IL‐10 induces or facilitates fusion or if FBGCs are formed by IL‐10‐producing macrophages remains unclear.

Although IL‐10 did not increase axon numbers in the bridge, IL‐10 robustly improved motor function after SCI **(**Figure 8 **)**. IL‐10 may act to moderate the deleterious immune response, resulting in improved axon sparing. Increased sparing with increased IL‐10 expression is consistent with previous reports, which have demonstrated that IL‐10 reduces loss of neurons and oligodendrocytes directly through trophic support^38^ and indirectly by limiting the immune response.^59^ Additionally, in our past work, differences in numbers of regenerating axons between conditions become more pronounced at later time points.^54^ Thus, improved motor function observed in the current study suggests improved sparing and plasticity instead of regeneration. While these data suggest that IL‐10 overexpression from PLG bridges effectively improves function after SCI, delivery of IL‐10 in combination with neurotrophic factors known to promote axon regeneration, such as neurotrophin‐3 (NT‐3),^54^, ^57^ may act synergistically to further improve both sparing and regeneration.

The present study indicates that immobilization of IL‐10 lentivirus onto multiple channel bridges alters the immune response to create a microenvironment that is permissive to regeneration and shows promise as a translatable strategy. The bridges used are made of PLG, a biomaterial that has been utilized for decades in FDA‐approved applications, including biodegradable sutures and drug delivery vehicles.^95^ PLG is easily sterilized for clinical using standard techniques, most commonly γ‐irradiation.^96^ Though safety has been cited as a concern for lentiviral vectors, there are ongoing clinical trials using lentivirus without adverse effects or evidence of insertional mutagenesis.^97^, ^98^, ^99^ Pyrogens from virus production can be limited by purification through gravity‐flow columns to yield endotoxin‐free, concentrated plasmid, as done in these studies. Furthermore, good manufacturing practices (GMP) have been developed for large‐scale preparation of lentivirus for clinical use.^98^, ^100^

Finally, the hemisection model of SCI, while not perfectly representative of all SCI, is a translatable platform for studying the injury site and developing treatments. While contusive injuries represent the majority of SCI in the developed world, at least 28% of cases in the US military are penetrating,^101^ and in South Africa, more than 60% of SCI is categorized as penetrating.^102^ Moreover, the initial deficits resulting from penetrating SCI exhibit substantially less improvement over time than those from contusive injuries.^103^ For more severe contusions, even if tissue architecture is initially preserved, secondary damage results in cavitation, loss of parenchyma, and glial scarring. Severe contusions and penetrating injuries may require a biomaterial bridge to provides a permissive pathway for regenerating neurons to cross the injury site and reconnect spinal pathways through true axonal regeneration, as opposed to local sprouting.^104^

## 5 Conclusions

We report that delivery of IL‐10‐encoding lentivirus from multiple‐channel bridges to reduce the innate immune response following injury. IL‐10 overexpression reduces PMN infiltration into the injured spinal cord compared to the delivery of control lentivirus. IL‐10 overexpression induced arginase expression in macrophages and substantially altered macrophage morphology. Additionally, IL‐10 lentivirus resulted in substantially improved motor function. This result indicates that IL‐10 will be an essential component of subsequent combinatorial gene therapies in order to reduce cell death and inhibition.
